# The oral–lung axis in lung cancer: microbial translocation, immune reprogramming, and clinical implications

**DOI:** 10.3389/fimmu.2026.1939497

**Published:** 2026-09-10

**Authors:** Xiangliang Liu, Guanran Ding, Jin Lu, Wei Song, Yan Li, Xiao Chen, Jiuwei Cui

**Affiliations:** Cancer Center, The First Hospital of Jilin University, Changchun, China

**Keywords:** lung cancer, microaspiration, microbial biomarkers, oral microbiome, oral-lung axis, periodontal disease, Th17 inflammation, tumor microenvironment

## Abstract

The oral–lung axis describes microbial and inflammatory exchange between the oral cavity and the lower respiratory tract. Its relevance to lung cancer is supported by the detection of oral-associated taxa in lower-airway samples, epidemiological links between periodontal disease and lung cancer risk, and experimental evidence that microbial signals can modify epithelial proliferation and antitumor immunity. This review integrates epidemiological, ecological, mechanistic, and translational evidence while distinguishing oral-associated or source-linked observations from pulmonary and intratumoral mechanisms in which oral origin has not been demonstrated. Particular attention is given to repeated microaspiration, Th17/IL-17 inflammation, NOD2/NF-κB and PI3K/ERK signaling, microbial metabolites, and remodeling of the tumor immune microenvironment. Recent studies of pulmonary nodules, radiotherapy, and immune checkpoint inhibition extend this field from disease association to clinically testable questions. The available evidence is most consistent with context-dependent tumor promotion and treatment-response modulation, rather than independent microbial initiation of human lung cancer. We conclude by defining the source-tracking, analytical, prospective, and interventional studies needed to establish causality and clinical utility.

## 1 Introduction

Lung cancer remains the leading cause of cancer mortality worldwide, accounting for approximately 1.8 million deaths in 2022 (1). Although advances in molecularly targeted therapy and immune checkpoint inhibition have improved outcomes for selected patients, population-level survival remains poor, particularly in advanced disease (2). Tobacco smoking continues to dominate lung cancer risk, alongside radon, occupational carcinogens and ambient air pollution. However, lung cancer also arises in never-smokers, in whom its epidemiological and molecular characteristics differ from those of smoking-related disease (3). This etiological diversity has broadened interest in biological factors that interact with inhalational exposures, epithelial injury and host immunity. The microbiome has become increasingly relevant within this framework, not necessarily as an independent carcinogen, but as a potential modifier of chronic inflammation and the pulmonary tumor microenvironment.

The lower respiratory tract was long considered sterile, largely because conventional culture methods were insufficiently sensitive to recover its sparse microbial communities. Culture-independent 16S rRNA sequencing and, subsequently, metagenomic approaches established that healthy lungs contain low-biomass but structured microbial communities (4–6). Their composition is governed by the balance among microbial immigration, regional growth conditions and elimination through mucociliary clearance, cough and innate immune defense. A substantial proportion of lower-airway bacteria originate from the upper aerodigestive tract, and healthy lung communities resemble oral and oropharyngeal microbiota more closely than microbial communities at other body sites (7, 8). This relationship is captured by the adapted island model of lung biogeography, in which the pulmonary microbiome represents a dynamic ecological state rather than a fixed resident community (9). Repeated microaspiration of saliva and oropharyngeal secretions provides the principal route of microbial immigration and occurs during sleep even in healthy individuals (10–12).

The equilibrium described by the adapted island model is readily disturbed. Periodontitis increases the oral burden of dysbiotic bacteria and inflammatory products (13, 14). Smoking alters oral microbial composition and, together with COPD and chronic airway injury, changes the ecological and clearance conditions of the lower respiratory tract (5, 15–18). These conditions can alter both the composition of microbial material entering the lower respiratory tract and its fate after arrival. In lung cancer, several studies have reported a shift of lower-airway communities towards oral-associated taxa, suggesting that oral microbial immigration may become more consequential when pulmonary homeostasis is disrupted (19–23). This interaction between oral dysbiosis, repeated microbial transfer, and an altered pulmonary niche provides the biological basis for examining the oral–lung axis in lung cancer development and progression.

Recent reviews have addressed complementary aspects of this field, including microbial variation across respiratory sample types, respiratory microbiome–immunity interactions, mechanisms and therapeutic opportunities within the oral–lung-axis framework, and the potential influence of the oral microbiome on treatment outcomes (24–30). Taken together, this literature leaves unresolved how microbial function should be interpreted across anatomically connected but ecologically distinct compartments. Oral-associated taxa detected in the lower airways may reflect repeated immigration, local ecological selection, or both, whereas intratumoral microbial activity does not necessarily indicate an oral origin. This uncertainty complicates efforts to determine whether microbial changes precede lung cancer, arise during tumor development, or modify subsequent progression and treatment response. Here, we trace evidence from oral ecology and microaspiration to lower-airway and tumor-associated changes, integrating epidemiological, mechanistic, and clinical findings while considering the limitations of low-biomass sampling. This synthesis aims to clarify when the oral–lung axis provides a biologically plausible framework for understanding lung cancer and where causal inference remains premature.

## 2 The oral-lung axis: anatomical and microbiological foundations

### 2.1 Composition and ecology of the oral microbiome

The oral cavity is one of the most densely colonized microbial habitats in the human body and contains approximately 700 identified bacterial taxa (31). Its microbial communities are spatially organized across the tongue dorsum, buccal mucosa, saliva, supragingival plaque and subgingival crevice, each of which provides distinct oxygen, nutrient and adhesion conditions. Accordingly, the oral microbiome is not a single uniform community. *Streptococcus*, *Veillonella*, *Prevotella*, *Neisseria*, *Haemophilus*, *Actinomyces* and *Fusobacterium* are commonly detected, but their relative abundance varies substantially among oral sites and individuals (31, 32). Periodontitis represents a community-level disruption of this ecological organization. Accumulation of subgingival biofilm and an altered host inflammatory response favor inflammophilic and proteolytic organisms, including *Porphyromonas gingivalis*, *Treponema denticola* and *Tannerella forsythia*, alongside a broader shift in microbial composition and function (13, 14). The resulting periodontal niche contains an increased burden of bacterial cells, virulence factors and inflammatory products. Saliva continuously disperses this material from dental and mucosal surfaces, making periodontal dysbiosis relevant not only to local tissue destruction but also to microbial exposure at anatomically connected sites.

### 2.2 The lung microbiome as an oral-influenced ecosystem

The healthy lung contains a low-biomass but structured microbial community. Its composition is determined by three ecological processes: immigration from the upper aerodigestive tract, elimination through mucociliary clearance and host defense, and selective growth under local conditions such as oxygen tension, pH, mucus composition and nutrient availability (6, 9, 33). Because microbial immigration and elimination generally exceed local replication in healthy airways, variation in the source communities and in airway clearance can produce substantial differences in pulmonary microbial composition.

Evidence for oral contribution emerged from studies of healthy lower airways. Segal et al. identified a community type enriched in supraglottic taxa, particularly *Prevotella* and *Veillonella*, which was associated with increased bronchoalveolar lymphocytes and a Th17-related inflammatory profile (7, 21). Bassis et al. subsequently showed that lower-airway communities in healthy individuals were more closely related to oral and upper-airway microbiota than to environmental sources (8). These findings established microaspiration as a major determinant of pulmonary microbial immigration. The lung microbiome nevertheless remains distinct from the oral microbiome because airway anatomy, mucociliary transport and local immune selection determine which organisms are retained or removed. It is therefore best viewed as an oral-influenced pulmonary ecosystem rather than a passive extension of the mouth.

### 2.3 Routes of oral microbial entry into the lower respiratory tract

**Figure 1:**
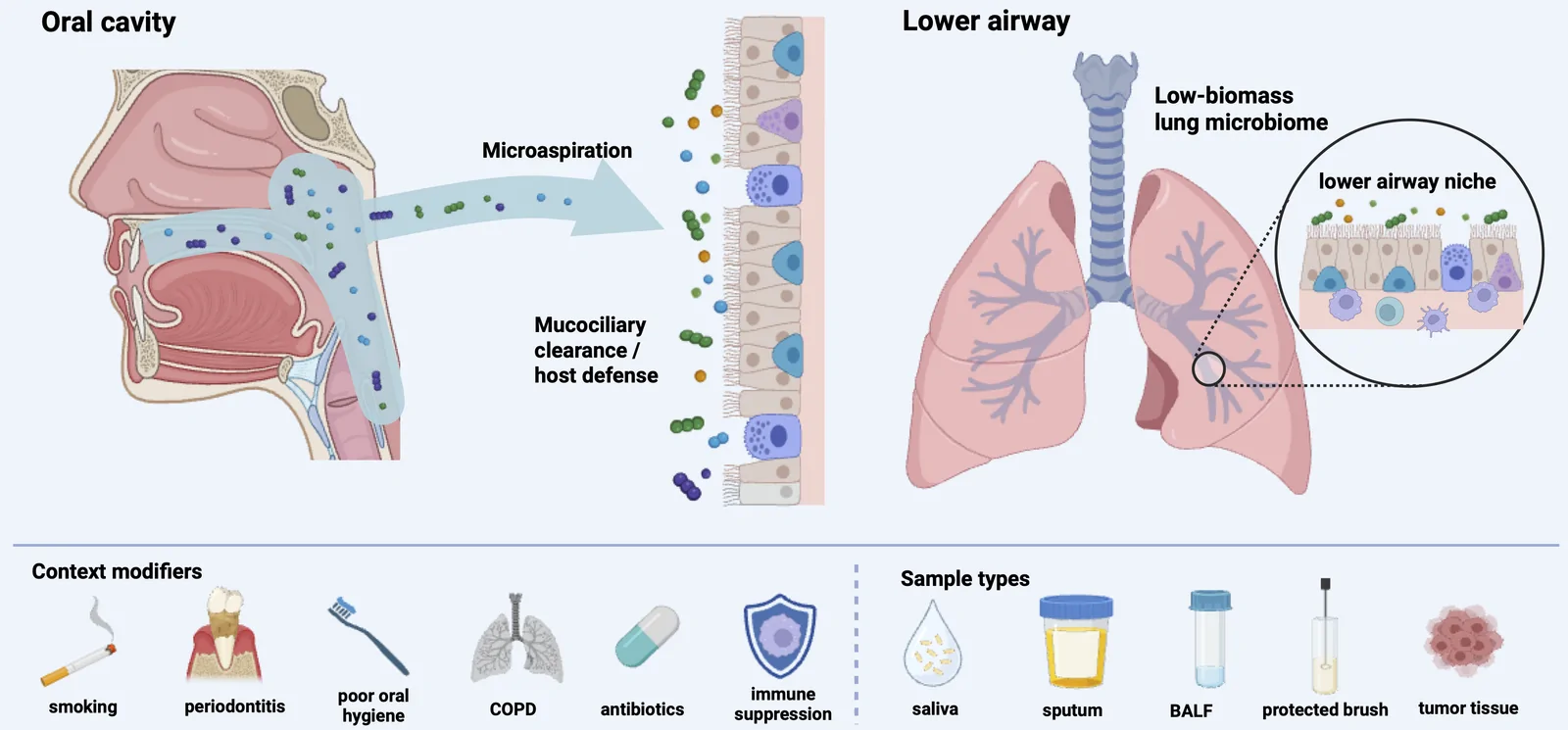
Anatomical and ecological framework of the oral–lung axis. Oral microorganisms and microbial products can enter the lower respiratory tract through microaspiration of oropharyngeal secretions. Their persistence in the low-biomass lung microbiome is determined by the balance between microbial immigration and elimination through mucociliary clearance and host defense. Smoking, periodontitis, poor oral hygiene, chronic obstructive pulmonary disease (COPD), antibiotic exposure and immunosuppression may alter oral microbial input, airway clearance or the local pulmonary environment. Common specimens used to investigate the oral–lung axis include saliva, sputum, bronchoalveolar lavage fluid (BALF), protected specimen brush samples and tumor tissue. These specimens represent different anatomical compartments and vary in invasiveness, microbial biomass and susceptibility to upper-airway contamination. Figures were created in BioRender.com and exported under a BioRender publication license.

Microaspiration is the best-supported route by which oral microorganisms reach the lower respiratory tract (**Figure 1**). Using radiolabeled tracers, Gleeson et al. detected aspiration of oropharyngeal secretions during sleep in nearly half of healthy participants, indicating that small-volume aspiration is a physiological event rather than an exclusively pathological process (12). Source-tracking studies subsequently showed that bacterial communities recovered from healthy lower airways are derived predominantly from oral and upper-airway sources (7, 8). For oral microorganisms, repeated microaspiration therefore provides a more direct explanation for lower-airway immigration than occasional large-volume aspiration.

Other routes are less clearly defined. Inhalation contributes environmental microorganisms to the respiratory tract, while contiguous dispersal across the upper-airway mucosa may contribute to proximal airway communities (6, 9, 33). However, their specific contribution to oral-to-lung transfer has not been quantified independently of microaspiration. Transient bacteremia arising from inflamed periodontal tissues provides another possible route, although direct evidence for hematogenous delivery of oral bacteria to human lung tissue remains limited (34). After reaching the lower airways, microbial persistence is constrained by mucociliary transport, cough, antimicrobial peptides, secretory immunoglobulins and phagocytosis by resident immune cells (6, 9). Pulmonary microbial composition consequently reflects both the rate of immigration and the efficiency of elimination. Alterations in either process can increase the duration and intensity of lower-airway exposure to oral-derived microorganisms without necessarily implying stable colonization.

### 2.4 Factors modifying oral microbial input and pulmonary persistence

Factors acting on the oral-lung axis can be organized according to whether they alter the oral source community, microbial transfer, or conditions within the recipient pulmonary niche. Periodontitis increases the biomass and inflammatory activity of subgingival communities and enhances the release of bacterial cells and products into saliva (13, 14). Smoking affects both ends of the axis: it reshapes oral microbial composition and impairs epithelial and mucociliary defenses within the respiratory tract (16–18). COPD and chronic airway injury further alter mucus properties, airway architecture and local immune selection, creating conditions that may prolong the persistence of aspirated organisms (5, 6).

Antibiotic exposure and immune suppression may also modify oral and pulmonary communities, although their direct effects on oral-to-lung microbial transfer have not been adequately quantified. These factors should therefore be treated as components of the host and exposure context rather than as independent translocation pathways. Their combined influence determines whether oral microorganisms are rapidly cleared after aspiration or remain sufficiently long to affect the lower-airway microenvironment (**Table 1**).

**Table 1:** Evidence domains and interpretive boundaries of the oral-lung axis in lung cancer.

Evidence domain	Representative evidence	Principal observation	What the evidence establishes	Main uncertainty	Key refs
Anatomical and ecological foundation	Paired oral, upper-airway and healthy lower-airway studies	Microaspiration continuously introduces oral and oropharyngeal microorganisms into the lower respiratory tract; pulmonary communities are then shaped by clearance and local selection.	Establishes biological plausibility and a source-to-recipient relationship between the oral cavity and lower airways.	Does not establish persistent colonization or a role in lung cancer.	(7–14, 21, 31–33)
Oral health epidemiology	Cohorts, nested case-control studies and meta-analyses of periodontitis and tooth loss	Periodontitis and cumulative oral tissue destruction are associated with higher lung cancer risk in several populations.	Supports a population-level association between oral disease burden and lung cancer.	Residual confounding by smoking, socioeconomic factors and oral-care access remains substantial.	(35–38)
Prediagnostic oral microbiome	Prospective oral-wash and salivary studies in smokers and never-smokers	Oral diversity and taxon-level profiles measured before diagnosis are associated with subsequent lung cancer risk.	Provides temporality for oral microbial variation and reduces concern about reverse causation.	Signals differ across cohorts; single baseline samples do not establish persistence or causality.	(39–41)
Lower-airway microbiome	BALF, protected brush and paired oral-lower-airway studies	Oral-associated community patterns recur in a subset of lung cancers and correlate with stage, progression and survival.	Links oral microbial input to local pulmonary ecology and clinically relevant disease states.	Most studies are cross-sectional and sensitive to sampling, oral carryover and low biomass.	(19–21, 51, 52)
Tumor-tissue microbiome	Resected tumors, adjacent lung and spatial transcriptomic analyses	Microbial signals vary by histology, TP53 status, smoking context and intratumoral location; selected metabolites correlate with recurrence or metastasis.	Supports context-dependent interactions within the tumor microenvironment.	Viability, absolute abundance, cellular localization and oral source attribution are often unresolved.	(45, 46, 49, 58)
Oral-associated mechanistic evidence	Human lower-airway transcriptomics, epithelial culture, and *V. parvula* cell and mouse models	Oral-enriched lower-airway communities associate with PI3K/ERK and Th17 programs; *V. parvula* activates NOD2/CCN4/NF-κB signaling and alters tumor burden and immune infiltration.	Supports functional plausibility that oral-associated microbial exposure can engage tumor-promoting epithelial and immune pathways.	Human profiles are largely genus-level; experimental exposure does not establish natural oral-to-lung transfer or dose.	(19–21, 54)
Pulmonary and intratumoral mechanistic evidence	Local lung-commensal perturbation, NTHi airway models, and intratumoral *Roseburia*/butyrate experiments	Local microbes drive MyD88-IL-1β/IL-23-γδT17, IL-17C-neutrophil, and butyrate-epigenetic/macrophage programs.	Shows that pulmonary or intratumoral microbial activity can promote tumor growth, immune remodeling, and metastatic behavior.	Oral origin was not tested or established; *Roseburia* is commonly gut-associated and its intratumoral source remains unresolved.	(55–58)
Boundary and negative evidence	Periodontitis Mendelian randomization and Sherlock-Lung never-smoker tissue analysis	Genetic liability to periodontitis was not associated with lung cancer, and no stable tissue or circulating microbial associations were identified in treatment-naive never-smokers within the detection and analytical framework used	Restricts broad causal claims and identifies population and compartment dependence.	Genetic liability is not measured exposure, and periodontitis is heterogeneous; the null tissue finding is population- and sampling-specific and does not exclude subgroup, spatial-niche, or low-abundance effects	(83, 84)
Methodological reliability	Low-biomass controls, contaminant modeling, MBQC, STORMS and analysis standards	Extraction, reagent contamination, batch structure and bioinformatic workflow can materially alter reported microbial profiles.	Defines the minimum quality criteria for interpreting lung microbiome studies.	Many published cohorts lack complete negative controls, absolute quantification or strain-level source tracking.	(47, 48, 85–88)

BALF, bronchoalveolar lavage fluid; ERK, extracellular signal-regulated kinase; IL, interleukin; MBQC, Microbiome Quality Control; NF-κB, nuclear factor κB; NOD2, nucleotide-binding oligomerization domain-containing protein 2; PI3K, phosphoinositide 3-kinase; STORMS, Strengthening the Organization and Reporting of Microbiome Studies; TP53, tumor protein p53.

## 3 Epidemiological evidence linking oral health to lung cancer

### 3.1 Periodontal disease and markers of cumulative oral disease

Epidemiological studies have repeatedly associated periodontal disease and poor oral health with lung cancer risk. A meta-analysis of six cohort studies and two case-control studies, comprising 167,256 participants, reported a pooled hazard ratio of 1.40 (95% CI, 1.25-1.58) in cohort studies and an odds ratio of 1.51 (95% CI, 1.16-1.98) in case-control studies (35). These estimates were derived from adjusted models reported by the individual studies and showed relatively low heterogeneity among the cohort analyses.

Evidence from the Southern Community Cohort Study further indicates that the association varies across population and exposure groups. In a nested case-control analysis of 403 incident lung cancers and 1,612 matched controls, a self-reported history of periodontal disease was associated with lung cancer among African American participants (OR, 1.56; 95% CI, 1.05-2.31) and heavy smokers (OR, 2.05; 95% CI, 1.38-3.05) (36). Loss of more than ten teeth was also associated with higher risk (OR, 1.64; 95% CI, 1.00-2.69), linking cumulative oral tissue destruction with lung cancer in the same population. Similar associations between periodontal disease, tooth loss and cancer have been reported in broader epidemiological analyses (37, 38).

### 3.2 Prediagnostic oral microbiome and lung cancer risk

Prospective microbiome studies have moved this field beyond self-reported oral disease. Vogtmann et al. examined prediagnostic oral-wash samples from 1,306 incident lung cancer cases across the Agricultural Health Study, NIH-AARP Diet and Health Study and PLCO Cancer Screening Trial (39). Higher oral microbial diversity was associated with lower lung cancer risk: each unit increase in the Shannon index corresponded to a hazard ratio of 0.90 (95% CI, 0.84-0.96). Several community components and individual taxa were also associated with risk; notably, each standard-deviation increase in *Streptococcus* abundance was associated with a 14% increase in lung cancer risk. Associations were more pronounced for squamous cell carcinoma and among former smokers.

A nested case-control study within the Southern Community Cohort Study also identified prediagnostic oral taxa associated with subsequent lung cancer, although it did not reproduce a significant difference in alpha diversity (40). This distinction is important: prospective cohorts support an association at the level of oral microbial composition, but the individual taxa and diversity measures carrying that association are not uniform across populations.

Smoking is not the only setting in which a prospective oral microbial signal has been observed. In 114 matched case-control pairs drawn from the Shanghai Women’s Health Study and Shanghai Men’s Health Study, all participants were lifetime never-smokers. Lower oral microbial diversity was associated with higher lung cancer risk, while several bacterial groups showed risk-associated abundance patterns in samples collected before diagnosis (41). These prospective data extend the temporal evidence to never-smokers by showing that oral microbial variation was detectable before diagnosis (41). A separate, hypothesis-generating line of evidence comes from a two-sample Mendelian randomization analysis reporting associations between genetically predicted oral microbial traits and lung cancer susceptibility (42). Because this approach estimates host-genetic proxies rather than measured microbial exposure, its interpretation is limited by instrument strength, population stratification, instability of taxonomic assignment, and horizontal pleiotropy.

## 4 Altered microbiome signatures in lung cancer

### 4.1 Microbial variation across sample types

Lung cancer microbiome studies have analyzed saliva, sputum, bronchoalveolar lavage fluid (BALF), protected specimen brush samples and tumor tissue. Saliva primarily reflects the oral microbiome and is well suited to repeated sampling and population-based studies. Sputum contains material from the oral cavity, upper respiratory tract and lower airways, providing a non-invasive respiratory sample without precise anatomical localization (26, 43, 44). BALF and protected brush specimens more closely represent the lower airways, with protected sampling offering greater anatomical specificity (22, 23). Tumor tissue provides direct access to microbial signals within the cancer niche, although its low microbial biomass makes the results particularly sensitive to tissue processing and reagent contamination (45–49).

Differences in microbial composition have been reported between patients with lung cancer and non-malignant controls across several sample types. The direction of change in alpha diversity, however, has varied among cohorts, with studies reporting lower, higher or unchanged diversity depending on the specimen, control population and analytical method (26, 50). The more reproducible observation is a shift in community composition rather than a uniform loss or gain of microbial diversity.

### 4.2 Oral-associated taxa in the lower airways

Paired oral and lower-airway sampling has provided direct evidence of microbial continuity between the two compartments. Sun et al. combined culturomics with 16S rRNA gene sequencing to characterize saliva and BALF from patients with pulmonary masses (51). They identified 198 bacterial species in BALF, 20 of which were cultured from at least half of the patients and were also abundant in oral samples. *Streptococcus* and *Veillonella* were among the most prominent cultured genera at both sites. The relative abundance of *Prevotella* and *Veillonella* declined from the oral cavity to BALF, whereas *Pseudomonas* increased in the lower airways, indicating that oral microbial input is modified by selection within the pulmonary environment (51). More recent paired profiling has also identified structural and functional differences across the upper and lower respiratory tracts in patients with NSCLC (52).

BALF and protected brush studies have further implicated oral-associated taxa in the restructuring of lower-airway communities in lung cancer. Lee et al. identified significant compositional differences in BALF between patients with lung cancer and those with benign mass-like lesions (22). Using bilateral protected specimen brushes, Liu et al. detected lung cancer-associated lower-airway microbial changes in patients with unilateral pulmonary masses (23). *Veillonella*, *Streptococcus* and *Prevotella* have appeared repeatedly across these studies, although the direction and magnitude of taxon-level changes have not been uniform among cohorts.

Tsay et al. subsequently examined the relationship between oral-enriched lower-airway communities and clinical features of lung cancer. Their initial study identified a lower-airway microbial pattern enriched in oral-associated genera, including *Streptococcus* and *Veillonella*, in patients with lung cancer (19). In a subsequent NSCLC cohort, this oral-enriched community type was more prevalent in patients with stage IIIB–IV disease (20). Among patients with stage I–IIIA disease, it was associated with poorer overall survival. In advanced disease, greater similarity between lower-airway and oral communities was associated with tumor progression assessed by RECIST (20). Oral enrichment in the lower airways therefore represents a clinically relevant ecological feature associated with disease stage and outcome.

### 4.3 Histological and molecular variation in the tumor tissue microbiome

Across cancer types, intratumoral microbial communities are increasingly recognized as tissue-specific components of the tumor ecosystem (53). In lung cancer, tumor-tissue microbial profiles appear particularly dependent on histological and molecular context. Greathouse et al. identified an association between microbial composition and TP53 mutation status in lung tumors. *Acidovorax* was enriched predominantly in TP53-mutant squamous cell carcinomas, whereas the same pattern was not observed in adenocarcinoma (45). Gomes et al. likewise reported distinct microbial profiles in adenocarcinoma and squamous cell carcinoma, with additional variation according to sex and smoking status (46). Analyses that combine lung cancer subtypes may therefore obscure microbial features confined to specific histological or molecular backgrounds.

Spatial metatranscriptomic analysis has added a further level of resolution. Wong-Rolle et al. found that intratumoral bacterial burden was unevenly distributed and associated with distinct oncogenic transcriptional states in adjacent lung cancer cells (49). This spatial relationship extends tissue microbiome analysis beyond whole-sample abundance profiles and indicates that microbial signals may be linked to localized cellular states within the tumor.

**Figure 2:**
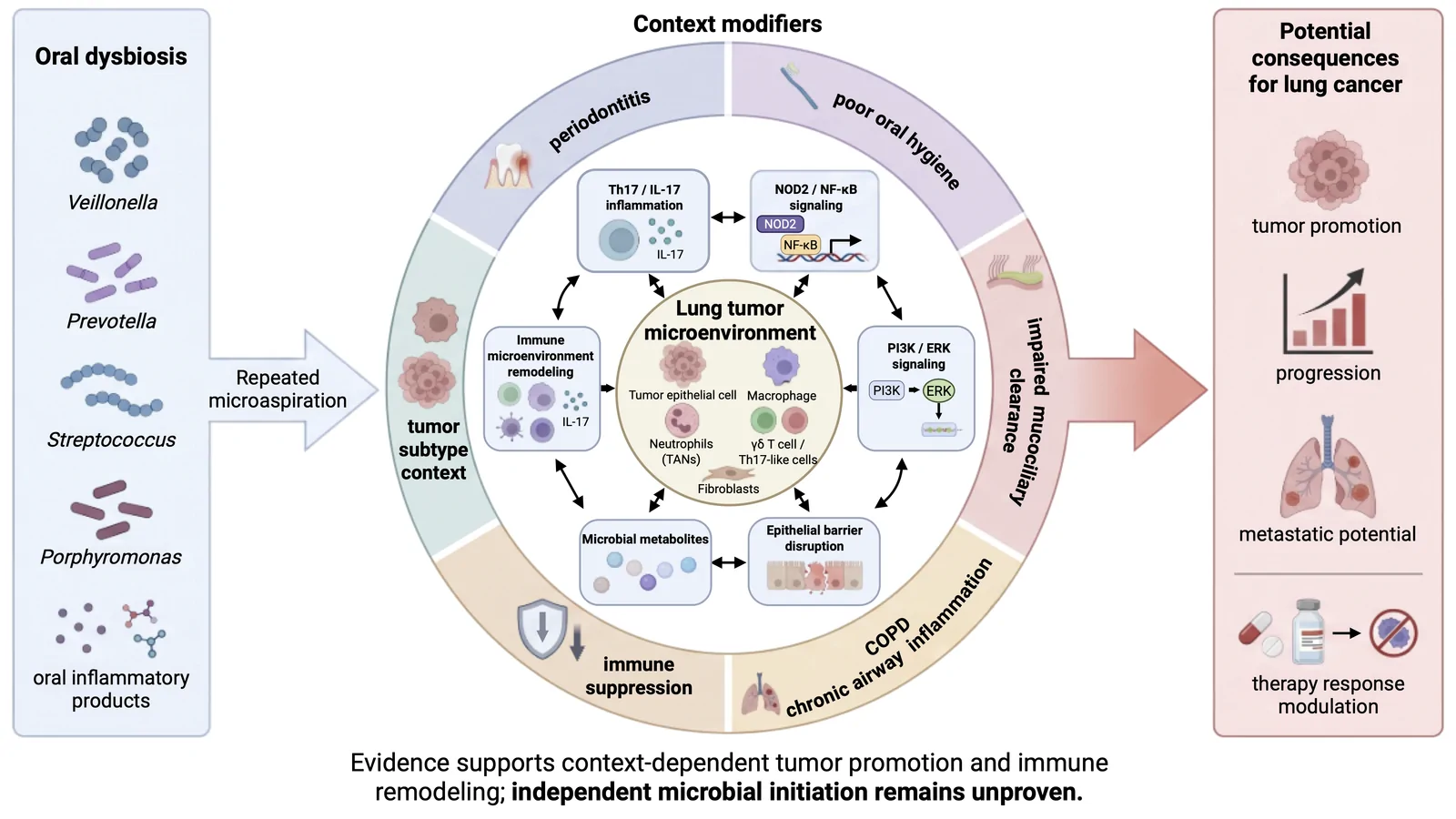
Context-dependent mechanisms linking oral dysbiosis to lung tumor promotion. Oral dysbiosis is characterized by altered community structure, increased inflammatory products, and shifts in oral-associated taxa, including *Veillonella, Prevotella, Streptococcus*, and *Porphyromonas*. Repeated microaspiration provides a plausible route by which oral microorganisms and their products may reach the lower airways. Their effects depend on host and disease context, including periodontitis, poor oral hygiene, impaired mucociliary clearance, COPD or chronic airway inflammation, immune suppression, and tumor-subtype context. Within the lung tumor microenvironment, microbial exposure may contribute to Th17/IL-17-associated inflammation, epithelial barrier disruption, microbial metabolite production, immune microenvironment remodeling, and activation of NOD2/NF-κB and PI3K/ERK signaling. These interacting processes may promote tumor growth and progression, influence metastatic potential, and modify treatment response. Current evidence supports a context-dependent tumor-promoting and immune-modulatory role; independent microbial initiation of human lung cancer has not been established. TANs, tumor-associated neutrophils. Figures were created in BioRender.com and exported under a BioRender publication license.

The available evidence thus points to two related but distinct patterns. BALF and protected brush studies identify enrichment of oral-associated taxa in the lower airways and link this ecological pattern to stage and clinical outcome. Tumor tissue studies instead emphasize variation according to histology, TP53 status, smoking background and intratumoral location. Lung cancer-associated microbial communities may therefore reflect the combined influence of oral microbial input, selection within the lower airways and the local tumor environment (**Figure 2**).

## 5 Mechanistic pathways: evidence for tumor promotion and immune remodeling

### 5.1 Activation of proliferative signaling in airway epithelial cells

Airway epithelial cells are among the first host cells exposed to oral microorganisms entering the lower respiratory tract. Tsay et al. combined 16S rRNA sequencing with airway epithelial transcriptomics in patients with lung cancer, benign pulmonary nodules, and healthy controls. Enrichment of the lower airways with oral-associated genera, particularly *Streptococcus* and *Veillonella*, was linked to increased ERK and PI3K signaling in the airway epithelium (19). A subsequent NSCLC cohort similarly showed increased PI3K-, MAPK-, and ERK-related transcriptional programs in oral-enriched lower-airway samples (20).

These observations were followed by functional studies in cell and animal models (**Supplementary Table S1**). *In vitro* exposure of airway epithelial cells to *Veillonella*, *Prevotella*, *Streptococcus*, or their bacterial products reproduced the pathway activation observed in clinical samples (19). At the species level, *Veillonella parvula* adhered to and invaded lung adenocarcinoma cells and promoted proliferation through NOD2/CCN4/NF-κB signaling (54). In mice, *V. parvula* exposure increased tumor burden and reduced T-lymphocyte infiltration within tumor tissue (54). This progression from patient samples to experimental perturbation supports a role for oral-associated bacteria in activating pro-proliferative epithelial signaling.

### 5.2 IL-17-dependent inflammation and immune remodeling

Oral-associated lower-airway communities have also been linked to a Th17-skewed mucosal immune state. In individuals without known pulmonary disease, Segal et al. identified a distinct inflammatory phenotype associated with supraglottic enrichment in BALF (21). Samples enriched with *Prevotella*, *Veillonella*, and other supraglottic taxa contained more Th17 cells, neutrophils, and lymphocytes, together with higher epithelial STAT3 expression and an altered inflammatory cytokine profile (21). In a mouse model of lung cancer, lower-airway exposure to *V. parvula* increased Th17 cells and PD-1-positive immune cells, extending the human observations into an experimental setting (20). The human and mouse findings support a model in which oral-associated lower-airway enrichment shifts local immunity toward a Th17-dominant, tumor-promoting state.

### 5.3 Local effects of pulmonary and intratumoral microbiota

Studies of pulmonary commensals, experimental airway colonization, and intratumoral microbiota add a local microenvironmental perspective. They primarily address how microorganisms act within the lung; their relationship to oral-to-lung migration remains unresolved because microbial sources were not traced. In mouse models of lung cancer, pulmonary commensals stimulated MyD88-dependent production of IL-1β and IL-23 by myeloid cells. This response activated IL-17-producing Vγ6^+^Vδ1^+^ γδ T cells and promoted local inflammation and tumor-cell proliferation (55). By contrast, antibiotic-induced depletion of commensal bacteria weakened γδT17 responses and pulmonary antitumor surveillance in a separate study (56). Chronic airway exposure to nontypeable *Haemophilus influenzae* activated epithelial TLR2/4–IL-17C signaling, increasing neutrophil recruitment and tumor growth (57). The effect of microbe-regulated IL-17 signaling is therefore not fixed, but varies with microbial context, tumor model, and local immune state.

Intratumoral microorganisms may also influence tumor progression through their metabolites. Tumors from patients with early postoperative recurrence showed enrichment of butyrate-producing bacteria, including *Roseburia* (58). Cell, patient-derived organoid, and mouse experiments indicated that butyrate enhanced invasion and metastasis through HDAC2 inhibition, increased H3K27 acetylation at the H19 promoter, and upregulation of H19. Butyrate exposure was also associated with M2-like macrophage polarization (58). These findings support a local intratumoral microbiota–metabolite pathway in lung cancer progression. Because *Roseburia* is generally regarded as a gut-associated genus and its intratumoral source was not established, this study does not define an oral-source pathway.

### 5.4 Periodontal barrier disruption as an upstream amplifier

Periodontal dysbiosis can increase the release of microorganisms and inflammatory products from the oral cavity by compromising epithelial barrier integrity. *Porphyromonas gingivalis* suppresses GRHL2 in oral epithelial cells, disrupting intercellular junctions and weakening the epithelial barrier (59). Its gingipains can also degrade junctional adhesion molecule 1, facilitating the passage of lipopolysaccharide and peptidoglycan through the gingival epithelium (60). These changes increase the opportunity for microbial cells and bacterial products to enter saliva or cross inflamed periodontal tissues.

Within the oral–lung axis, periodontal barrier failure is most relevant as an upstream determinant of the microbial and inflammatory material repeatedly delivered to the lower airways. Bacteria, lipopolysaccharide and peptidoglycan reaching the respiratory tract may provide repeated input to pattern-recognition receptors, epithelial growth pathways and IL-17-associated inflammatory circuits. Periodontitis may therefore amplify pulmonary microbial exposure without requiring *P. gingivalis* itself to reproduce the same barrier-disrupting effects in the lung. *P. gingivalis* has also been implicated in oral and orodigestive carcinogenesis (61, 62), but direct evidence that it persists in the lower airways or contributes to primary lung cancer remains lacking.

## 6 Clinical and translational implications

### 6.1 Non-invasive microbial biomarkers: from case-control detection to pulmonary nodule risk refinement

**Figure 3:**
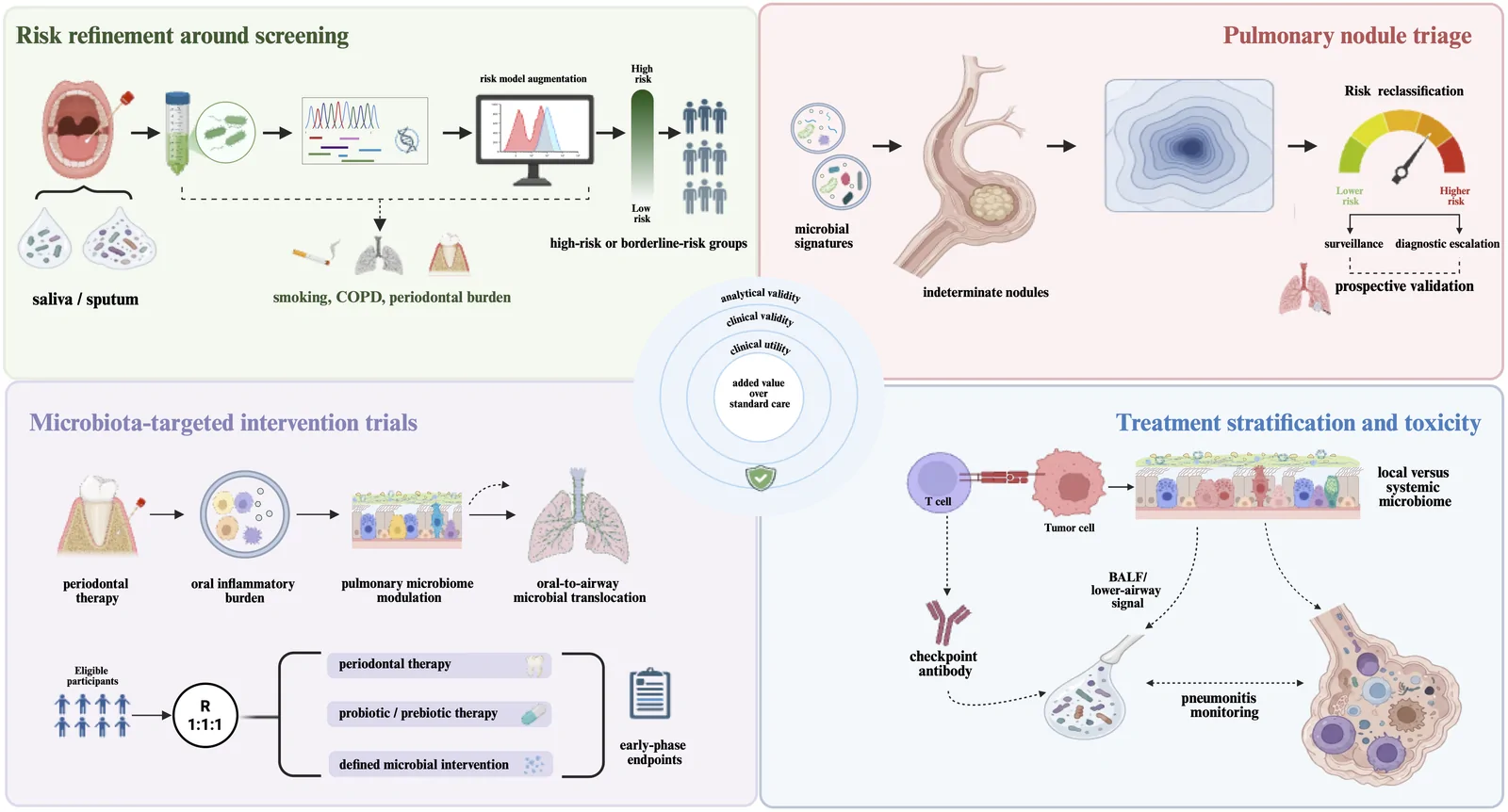
Clinical translation framework for the oral–lung axis in lung cancer. Potential clinical applications of oral and lower-airway microbial profiles span four areas. First, salivary or sputum microbial signatures may complement established risk factors, including smoking, COPD and periodontal burden, to refine risk assessment around lung cancer screening. Second, microbial classifiers may support risk reclassification of indeterminate pulmonary nodules and inform surveillance or diagnostic escalation, subject to prospective validation. Third, microbiota-targeted intervention studies may evaluate periodontal treatment, probiotic or prebiotic approaches and defined microbial interventions using oral inflammatory burden, lower-airway microbial changes and other early-phase outcomes. Fourth, local airway and systemic microbial profiles may be investigated in relation to immune checkpoint inhibitor response and treatment-related pulmonary toxicity, including checkpoint inhibitor pneumonitis. Across all applications, analytical validity and clinical validity must precede demonstration of clinical utility and measurable added value over standard care. BALF, bronchoalveolar lavage fluid; COPD, chronic obstructive pulmonary disease. Figures were created in BioRender.com and exported under a BioRender publication license.

Saliva and sputum are the most accessible specimens for clinical investigation of the oral–lung axis, whose principal translational opportunities are summarized in **Table 2** and **Figure 3**. Early case-control studies identified salivary signatures containing genera such as *Capnocytophaga* and *Veillonella* that distinguished patients with lung cancer from healthy controls (43). Metagenomic analysis of sputum likewise detected species-level profiles associated with lung cancer (44), while salivary dysbiosis has also been reported in non-smoking women with lung cancer (63). These studies established that lung cancer-associated microbial signals can be detected non-invasively, although their designs primarily addressed discrimination between established cancer and health rather than performance in a screening population.

**Table 2:** Translational opportunities and evidence requirements for the oral-lung axis.

Clinical use case	Specimen or intervention	Current evidence	Current maturity	Minimum evidence required for translation	Key refs
Screening-risk refinement	Saliva or sputum collected before imaging	Case-control studies distinguish established lung cancer from healthy controls, but do not demonstrate benefit in an LDCT-eligible population.	Discovery/association	Prospective sampling in screening cohorts; prespecified classifier; comparison with validated clinical risk models; calibration and decision-curve analysis.	(43, 44, 63, 64)
Pulmonary nodule triage	Saliva, oral rinse or lower-airway samples	Recent cohorts report microbial classifiers for persistent or indeterminate nodules and associations with malignant progression.	Early clinical validation	Locked model; blinded external validation; direct comparison with nodule size, morphology and established clinical-imaging models; net reduction in unnecessary procedures.	(65–68)
Prognostic and longitudinal monitoring	Saliva, BALF or tumor tissue collected at baseline or serially	Microbial features correlate with stage, survival, recurrence and radiotherapy response, but most models are small and treatment-specific.	Discovery/association	Serial sampling; clinically defined time points; validation by histology and treatment; incremental value beyond stage, tumor burden and molecular markers.	(20, 58, 71, 72)
ICI response stratification	Salivary, lower-airway and gut microbiomes	Baseline microbial and metabolic features correlate with ICI outcome; taxon-level signals vary by treatment regimen and anatomical compartment.	Discovery/association	Regimen-specific multicenter validation; standardized sampling; adjustment for antibiotics and corticosteroids; added value beyond PD-L1 and tumor burden.	(69, 70, 73, 75, 77, 78)
ICI toxicity monitoring	Lower-respiratory samples before and during treatment	Lower-respiratory microbial features have been associated with checkpoint inhibitor pneumonitis.	Discovery/association	Pre-treatment risk model; serial sampling before symptoms; adjudicated toxicity; comparison with radiological and inflammatory predictors.	(74)
Perioperative oral management	Periodontal assessment, professional cleaning and structured oral hygiene	Periodontal treatment reduces systemic inflammatory markers, and structured perioperative oral care is associated with fewer postoperative pulmonary complications.	Clinical association/implementation evidence	Prospective controlled implementation with standardized oral intervention, adherence assessment and predefined pulmonary complication endpoints.	(80, 81)
Microbiota-directed intervention	Targeted pulmonary delivery or defined microbial modulation	Aerosolized antibiotics or probiotics altered pulmonary communities and antitumor immunity in metastatic mouse models, although the affected communities were not linked to an oral source; systemic antibiotics are associated with poorer ICI outcomes.	Preclinical	Defined microbial target and mechanism; local pharmacodynamic and immune readouts; safety and ecological recovery; source tracking when an oral–lung mechanism is claimed.	(79, 82)
Assay qualification and clinical utility	Standardized collection, absolute quantification and contamination-aware sequencing	Low biomass, batch effects and pipeline variation remain major barriers to reproducible clinical classification.	Translational prerequisite	Analytical validity, reproducibility across laboratories, external validation, prospective clinical-utility trial and evidence of added value over standard care.	(47, 48, 85–88)

BALF, bronchoalveolar lavage fluid; CCN4, cellular communication network factor 4; ERK, extracellular signal-regulated kinase; IL, interleukin; MBQC, Microbiome Quality Control; MyD88, myeloid differentiation primary response 88; NF-κB, nuclear factor κB; NOD2, nucleotide-binding oligomerization domain-containing protein 2; NTHi, nontypeable *Haemophilus influenzae*; PI3K, phosphoinositide 3-kinase; STORMS, Strengthening the Organization and Reporting of Microbiome Studies; Th17, T helper 17; TP53, tumor protein p53; γδT17, interleukin-17-producing γδ T cells.

Maturity definitions: preclinical, evidence restricted to experimental models; discovery/association, association or model derivation without independent validation in the intended-use population; early clinical validation, evaluation in the intended-use population with limited external validation; clinical association/implementation evidence, observed clinical outcomes without definitive proof of intervention efficacy or clinical utility; translational prerequisite, a cross-cutting requirement that must be satisfied before any use case can progress to prospective utility testing.

Low-dose computed tomography (LDCT) remains the evidence-based basis of lung cancer screening (64). A more defined role for microbial biomarkers may lie in the assessment of pulmonary nodules detected by screening or incidental imaging. In MCEPN-1, salivary microbiota were profiled in 173 patients with pulmonary nodules and 40 healthy controls.

A five-genus classifier comprising *Fusobacterium*, *Porphyromonas*, *Parvimonas*, *Peptostreptococcus* and *Haemophilus* achieved an area under the curve of 0.80 for distinguishing the two groups (65). Because the comparator was a healthy population, this model identifies the presence of pulmonary nodules rather than their malignant potential. Subsequent studies have addressed the more clinically relevant distinction between benign and malignant nodules. Salivary and oral microbial profiles have been evaluated in persistent and indeterminate pulmonary nodules, including a prospective multicenter study, while enrichment of oral taxa in the lower respiratory tract has been associated with malignant progression (66–68). Clinical relevance will depend on whether a locked microbial classifier improves malignancy prediction beyond nodule size, morphology, smoking exposure and established clinical-imaging models. Microbiome testing would then serve as an adjunct to imaging-based assessment rather than as an alternative to LDCT.

### 6.2 Microbial correlates of treatment response and toxicity

Salivary microbiome studies have produced treatment-specific associations in patients receiving immune checkpoint inhibitors. In a cohort of advanced NSCLC treated with anti-PD-1 or anti-PD-L1 monotherapy, higher baseline salivary *Actinomyces* abundance was independently associated with shorter progression-free and overall survival (69). A smaller cohort receiving chemo-immunotherapy showed the opposite pattern: *Actinomyces* was more abundant among responders and was linked to lipid metabolites associated with treatment efficacy (70). Differences in treatment regimen, cohort size and metabolic context may therefore alter the clinical meaning of the same bacterial genus. These findings favor multivariable, treatment-specific microbial signatures over a universal single-taxon marker.

Shi et al. longitudinally profiled oral-rinse samples collected before and after radiotherapy from 24 patients with lung cancer; publicly available datasets comprising an additional 112 cases and 161 healthy controls were used to validate the diagnostic signature (71). In the 24-patient discovery cohort, a two-species model based on *Prevotella salivae* and *Neisseria oralis* yielded an area under the curve of 0.74 for distinguishing responders from non-responders. *Rothia aeria* and *P. salivae* were enriched among responders and associated with longer survival (71). Because the response model was derived from 14 responders and 10 non-responders and was not externally validated, its predictive performance remains preliminary. In resectable NSCLC, full-length 16S rRNA sequencing of preoperative saliva from 64 patients identified lower diversity in stage III than in stage I disease and stage-associated differences in *Haemophilus* and *Solobacterium* (72). The latter study supports perioperative prognostic research but did not test a microbiome-guided treatment strategy.

Lower-airway samples may capture microbial and metabolic states closer to the pulmonary tumor environment. Integrated 16S rRNA sequencing and metabolomic analysis of BALF from 26 patients with advanced NSCLC receiving ICI monotherapy identified community-level and metabolic differences associated with treatment response (73). Lower-respiratory microbial features have also been associated with checkpoint inhibitor pneumonitis (74). These findings remain exploratory, and their incremental value over PD-L1 expression, tumor burden, treatment regimen and established clinical predictors has not been determined.

Gut microbiome studies provide complementary evidence that microbial communities and antibiotic exposure can influence ICI outcomes (75–79). Their relevance lies primarily in systemic immune regulation, whereas saliva and lower-airway samples interrogate microbial compartments more directly connected to the pulmonary tumor environment. These compartments should therefore be evaluated in parallel rather than treated as interchangeable sources of a single microbiome biomarker.

### 6.3 Microbiota-directed intervention: perioperative oral care and pulmonary modulation

Perioperative oral management currently provides the most direct clinical evidence for intervention at the oral–lung interface. In patients undergoing pulmonary resection, a structured program comprising oral hygiene instruction, periodontal assessment, scaling, professional mechanical tooth cleaning and removal of tongue coating was associated with lower rates of postoperative pneumonia and Clavien–Dindo grade II or greater complications, as well as a shorter postoperative hospital stay (80). These outcomes are clinically relevant to thoracic surgery and do not depend on assuming an anticancer effect of oral treatment.

Periodontal therapy may also reduce inflammatory exposure beyond the oral cavity. A meta-analysis of randomized clinical trials showed that periodontal treatment lowered circulating C-reactive protein (81). This finding provides evidence that modifying periodontal inflammation can affect systemic inflammatory status. It does not establish that periodontal treatment reduces lung cancer incidence, recurrence or resistance to systemic therapy. Studies in patients with lung cancer should therefore distinguish perioperative complication prevention from modification of tumor-related outcomes.

Direct manipulation of the pulmonary microbiome remains preclinical. In mouse models of lung metastasis, aerosolized antibiotics or probiotics altered pulmonary microbial communities and enhanced local antitumor immune surveillance (82), providing proof of principle for a pulmonary microbiota–immune interaction. Because the affected communities were not linked to an oral source, this work does not by itself establish an oral–lung intervention. The metastatic models do not establish efficacy in primary human lung cancer, and clinical associations between systemic antibiotic exposure and poorer ICI outcomes caution against non-selective microbial depletion (79, 82). Any pulmonary microbiome intervention will need to define its microbial target, delivery route, and local immune consequences before clinical evaluation.

## 7 Challenges, controversies, and future directions

### 7.1 Causal direction and population boundaries

A central question is where microbial changes occur in the sequence of lung cancer development and progression. Current observations are compatible with several processes. Oral-derived microorganisms may alter pulmonary inflammation and immunity after reaching the lower airways; an established tumor may change local oxygen tension, nutrient availability and immune activity, thereby selecting a different microbial community; or shared factors such as smoking, COPD, antibiotic exposure and periodontitis may affect both cancer risk and microbiome composition. Case-control studies cannot readily distinguish among these possibilities. Prediagnostic oral samples provide stronger temporal evidence (39–41), but a single baseline measurement does not establish the persistence of a microbial feature or identify when it emerged relative to tumor development.

A Mendelian randomization analysis found no association between genetic liability to periodontitis and lung cancer risk (83). This finding constrains a broad causal interpretation of periodontitis but does not directly address actual oral microbial exposure. Given the phenotypic heterogeneity of periodontitis, it cannot exclude potential effects of specific microbial strains, microbial metabolites, or lower-airway ecological alterations.

The Sherlock-Lung study provides a complementary boundary for tissue-based microbiome associations. Using 16S rRNA gene sequencing, whole-genome sequencing, and RNA sequencing, the study analyzed 4,090 tumor, adjacent normal lung, and blood samples from 940 treatment-naive never-smokers with lung cancer (84). Within the detection and analytical framework used, no stable associations were identified between tissue or circulating microbial profiles and clinical or genomic features. This large negative study therefore defines an important population- and sampling-specific boundary, but it does not demonstrate the absence of locally active microorganisms in lung tumors. The samples represent a cross-sectional snapshot at surgical resection, and the study does not exclude effects confined to specific patient subgroups or spatial niches, particular low-abundance or culturable strains, or microbial changes emerging during disease progression or treatment. Nor does it address prediagnostic oral microbial variation or lower-airway dysbiosis in smoking-related lung cancer.

Lung cancer is unlikely to represent a single microbial ecological setting. Smoking-related airway injury, COPD or emphysema, periodontal burden, histological subtype and oncogenic drivers may each influence microbial immigration, clearance and local selection. These variables should be incorporated into cohort design and prespecified stratification rather than treated solely as covariates in pooled analyses.

### 7.2 Low-biomass measurement, contamination, and source attribution

Microbial biomass in lung tissue and BALF is substantially lower than in oral or intestinal samples. Small amounts of bacterial DNA introduced through reagents, laboratory environments or sample processing can materially alter community profiles and may produce apparently reproducible differences that do not originate from the biological specimen (47, 48). Negative controls, extraction blanks, positive controls, sample randomization and batch information therefore need to be processed and analyzed alongside biological samples. Frequency- and prevalence-based contaminant detection can further distinguish low-abundance biological signals from laboratory background (85).

Relative abundance introduces a separate interpretive problem. An increase in the proportion of one taxon does not establish that its absolute abundance has increased; the same pattern may result from depletion of other community members. Measurements of total bacterial burden, quantitative PCR or other absolute quantification approaches should accompany compositional analyses where possible. The Microbiome Quality Control project demonstrated that DNA extraction, amplicon selection, sequencing platform and bioinformatic workflow can substantially alter the taxonomic profile obtained from the same microbial material (86). STORMS and established microbiome-analysis recommendations provide practical standards for study design, quality control, analysis and reporting (87, 88).

Detection of a common oral genus in BALF is not equivalent to demonstrating oral origin. Source attribution requires paired oral, lower-airway and, where available, tumor samples from the same patient, followed by comparison at strain or genome resolution. Culturomics can establish the presence of viable organisms, whereas strain-resolved sequencing can determine whether isolates from separate compartments are closely related (51). Combined with absolute quantification, these approaches can distinguish repeated immigration from prolonged persistence or local expansion within the pulmonary niche.

### 7.3 From taxonomic association to functional and spatial evidence

Differences in relative abundance establish that microbial signals occur in association with lung cancer, but they do not demonstrate biological activity. Functional interpretation requires evidence that the organisms are viable or transcriptionally active, that they produce relevant metabolites, and that they interact with defined host-cell populations. Culture, metagenomics, metatranscriptomics and metabolomics address different aspects of this problem and cannot be substituted by taxonomic inference from 16S rRNA sequencing alone.

Spatial localization is particularly important in tumor tissue. Bacterial signals within tumor cells, macrophages, stromal structures or necrotic regions are unlikely to have the same biological significance. Spatial metatranscriptomic analysis has linked intratumoral bacterial burden with distinct transcriptional states in adjacent lung cancer cells (49). Studies in other cancers have also demonstrated intracellular bacterial localization and regional organization within tumors (89, 90). Combining *in situ* hybridization, imaging or spatial omics with culture and metabolic measurements would help distinguish residual microbial DNA, bystander organisms and locally active microbial populations (49, 91).

Most oral–lung axis studies remain centered on bacteria and 16S rRNA sequencing. The oral mycobiome, virome and cross-kingdom interactions have received comparatively little attention in lung cancer. Pan-cancer analyses have identified cancer-type-specific fungal ecologies and relationships between fungal and bacterial communities (92). Restricting microbiome analysis to bacteria may therefore overlook ecological interactions that influence mucosal inflammation, microbial persistence or antitumor immunity.

### 7.4 Study designs aligned with etiological and clinical questions

Etiological studies need to determine whether oral microbial changes precede lung cancer and remain stable over time. Repeated collection of prediagnostic saliva or oral-rinse samples in multi-ethnic prospective cohorts would allow persistent microbial features to be distinguished from short-term variation. These studies should include clinical periodontal assessment and detailed information on smoking dose and duration, second-hand smoke, antibiotic exposure, COPD, lung function and imaging findings (39, 40). Incident cancers should be analyzed by histological and molecular subtype rather than combined into a single endpoint.

Mechanistic studies require a direct link between human source evidence and controlled experimental systems. Paired oral, BALF and tumor samples can support strain-level source tracking; culture and activity measurements can identify organisms with functional potential; and spatial methods can determine their proximity to epithelial, immune and stromal cells (49, 51). Candidate strains or defined microbial communities can then be tested in organoids, gnotobiotic systems or humanized models for effects on epithelial signaling, IL-17-dependent inflammation, metabolite production and immune regulation (54–58). This sequence provides a more defensible route from human microbial association to experimental perturbation than selecting taxa on relative abundance alone.

Clinical studies should begin with a prespecified use case. Pulmonary nodule studies need locked microbial classifiers, independent validation and direct comparison with nodule morphology, smoking history and established clinical-imaging models (65–68). Treatment-response studies require serial sampling before and during therapy and should determine whether microbial features add information beyond PD-L1 expression, tumor burden and treatment regimen (69–74). Initial intervention studies can use postoperative pneumonia, periodontal inflammation, systemic inflammatory markers and lower-airway microbial changes as measurable endpoints (80, 81). Direct manipulation of the pulmonary microbiome will require clear microbial targets, defined delivery methods and evidence of local immune effects before evaluation in patients (82).

## 8 Conclusions

The oral–lung axis connects periodontal dysbiosis and repeated microbial immigration with ecological and host changes in the lower respiratory tract. Human studies consistently identify oral-associated taxa in a subset of lower-airway communities, while experimental work shows that microbial exposure can engage epithelial growth signaling, IL-17-dependent inflammation, immune remodeling and metabolite-mediated tumor progression. The available evidence supports a context-dependent role in tumor promotion and progression, shaped by smoking-related airway injury, periodontal burden, impaired clearance and tumor subtype. It does not support a universal microbial pathway or establish oral microorganisms as independent initiators of human lung cancer.

The most credible translational opportunities lie in pulmonary nodule risk refinement, treatment-response and toxicity assessment, and perioperative oral management. Progress in these areas will depend on longitudinal paired sampling, strain-resolved source tracking, functional and spatial validation, and standardized analysis of low-biomass specimens. Clinical adoption will require prospective evidence that microbial measurements improve decisions beyond established imaging, pathological and treatment biomarkers. Framed in this way, the oral–lung axis provides a focused model for investigating how oral microbial exposure modifies lung cancer biology and clinical behavior.
