# Supplementary material for: The oral–lung axis in lung cancer: microbial translocation, immune reprogramming, and clinical implications
Source: Front Immunol. 2026 Sep 10;17:1939497. doi: 10.3389/fimmu.2026.1939497 (PMC13601903; doi:10.3389/fimmu.2026.1939497)
Supplement: Supplementary file 1 [file DataSheet1.pdf]

Supplementary material

Supplementary Methods: Literature Identification and Evidence Selection

PubMed was searched from database inception to 30 June 2026. Search terms covered the oral and salivary microbiomes, periodontitis, microaspiration, the oral–lung axis, the lung and lower-airway microbiomes, the intratumoral microbiome, lung cancer, and non-small cell lung cancer (NSCLC). Focused supplementary searches addressed diagnostic and treatment outcomes, microbiome-directed interventions, and methodological issues related to low-biomass samples. Additional studies were identified through reference-list screening and forward citation tracking of relevant primary studies and recent reviews.

Records were screened by title and abstract, followed by full-text assessment of potentially eligible articles. Eligible publications included peer-reviewed original studies directly relevant to oral health or oral, lower-airway, and intratumoral microbial communities in relation to lung cancer risk, progression, or treatment response. Human observational and clinical studies, in vitro and organoid experiments, and animal models were considered. Reviews were used to define the scope of the field and identify relevant original studies. Given the heterogeneity in specimen types, analytical methods, study designs, and outcomes, the evidence was synthesized narratively. Negative findings and studies defining the boundaries of causal interpretation were also retained. This review is narrative rather than systematic: literature identification was purposive and intended to represent the principal lines of evidence relevant to the oral–lung axis rather than to achieve exhaustive retrieval, and reference-list screening and forward citation tracking were used to reduce the likelihood of omitting influential primary studies.

Supplementary Table S1. Mechanistic evidence stratified by study design and source attribution

Mechanistic theme	Human evidence	In vitro or organoid evidence	In vivo evidence	Source attribution	Interpretive scope
Epithelial PI3K/ERK signaling	Oral-enriched lower-airway communities associated with epithelial PI3K/ERK programs (19, 20).	Airway epithelial exposure to oral-associated taxa or bacterial products reproduced pathway activation (19).	Not assessed in the cited studies.	Oral-associated genera identified by paired oral and lower-airway sampling; community-level, not strain-resolved.	Human association with in vitro functional support.
Veillonella parvula-NOD2/CCN4/NF-κB signaling	No direct human source-tracking experiment.	*V. parvula* adhered to and invaded lung adenocarcinoma cells and promoted proliferation (54).	Experimental exposure increased tumor burden and altered T-cell, Th17, and PD-1-positive immune states (20, 54).	Defined oral-associated species (*V. parvula*) introduced experimentally; natural oral-to-lung transfer not tested.	Functional capacity of a defined oral-associated species in experimental systems.
Th17 and γδT17 immune remodeling	Paired-compartment studies linked supraglottic enrichment in BALF to Th17-skewed inflammation (21).	No stand-alone cell model central to the cited evidence.	Lung-commensal perturbation implicated MyD88-IL-1β/IL-23-γδT17 signaling in tumor-promoting inflammation (55), whereas antibiotic depletion of commensals weakened γδT17 responses and pulmonary antitumor surveillance (56).	Human data: oral-associated lower-airway communities. Mouse data: resident lung commensals, source not traced.	Convergent human association and in vivo pulmonary mechanism, with opposing directions across models and differing source certainty.
IL-17C-neutrophil inflammation	No corresponding oral-lung human cohort in the cited study.	Bacterial stimulation identified epithelial TLR2/4-IL-17C signaling (57).	Chronic nontypeable *Haemophilus influenzae* (NTHi) airway exposure increased neutrophil recruitment and tumor growth (57).	Airway colonization model; oral origin not tested.	Pulmonary bacterium-inflammation-tumor mechanism.
Intratumoral butyrate signaling	Early-recurrence tumors showed enrichment of butyrate-producing bacteria, including *Roseburia* (58).	Butyrate promoted migration and invasion in cancer cells and patient-derived organoids (58).	Butyrate-producing bacteria or butyrate enhanced tumor growth and metastatic activity (58).	Intratumoral bacteria, source not traced; *Roseburia* is commonly gut-associated.	Multimodal evidence for a local microbiota-metabolite-metastasis pathway, not oral source attribution.
Periodontal barrier disruption	Periodontitis provides the clinical upstream context; direct lung-cancer causality remains unresolved.	*Porphyromonas gingivalis* disrupted GRHL2-dependent junctional integrity and gingipains degraded JAM1 in oral epithelium (59; 60).	No cited lung-cancer animal model directly tests this upstream route.	Oral compartment only; downstream pulmonary delivery not directly demonstrated.	Upstream amplification mechanism within the oral compartment.

Abbreviations: BALF, bronchoalveolar lavage fluid; CCN4, cellular communication network factor 4; ERK, extracellular signal-regulated kinase; GRHL2, grainyhead-like transcription factor 2; IL, interleukin; JAM1, junctional adhesion molecule 1; MyD88, myeloid differentiation primary response 88; NF-κB, nuclear factor κB; NOD2, nucleotide-binding oligomerization domain-containing protein 2; NTHi, nontypeable *Haemophilus influenzae*; PD-1, programmed cell death protein 1; PI3K, phosphoinositide 3-kinase; Th17, T helper 17; TLR, Toll-like receptor; γδT17, interleukin-17-producing γδ T cells.
